# Ultrasonographic, endoscopic and radiographic examinations of the dromedary mammary glands and teats

**DOI:** 10.1007/s11250-024-04009-8

**Published:** 2024-05-31

**Authors:** Müller Annika, Ulrich Wernery, Jörg Kinne, Peter Nagy, Judit Juhasz, Matthew De Bont, Claire Booth, Panagiotis Azmanis, Thomas Wittek

**Affiliations:** 1 Vetmeduni Vienna, University Clinic for Ruminants, Veterinärplatz 1, 1210 Vienna, Austria; 2 Central Veterinary Research Laboratory/CVRL, P.O. Box 597, Dubai, UAE; 3 Emirates Industry for Camel Milk Products, Dubai, UAE; 4 Dubai Camel Hospital, Al Marmoon, Al Ain, Dubai Road, Dubai, UAE; 5 Dubai Falcon Hospital, Al Saada St, Zaabeel, Zaabeel 2, Dubai, UAE

**Keywords:** Dromedary, Udder, Teat, Ultrasonography, Endoscopy, Radiography

## Abstract

The aim of the present study was to examine the mammary gland of dromedary camels using ultrasonography, endoscopy and radiography. These techniques are easy to perform in the field and feasible to diagnose pathological conditions of the mammary gland. Udders of 49 slaughtered and 26 adult dromedary camels submitted for necropsy were used for the examinations. Additionally, 11 lactating female dromedary camels were selected for the ultrasonographic udder examination. The transition from the milk ducts into the udder cistern, the teat cistern and the teat canals were examined in individual udders. Teat cistern length, teat end width, teat wall thickness, teat cistern width and middle cistern wall thickness were measured using ultrasonography. The measurements resulted in mean values of the teat cistern length of 37.3 mm, the teat end width of 2.0 mm, the teat wall thickness of 4.4 mm, the teat cistern width of 8.2 mm and the cistern wall thickness of 3.5 mm. The teat wall was differentiated into three layers, a hyperechoic outer layer, a hypoechoic middle layer and a hyperechoic inner layer. The mid cistern wall was hyperechoic. Endoscopic examination is an easy to perform and practicable method for examining the inner structures of the teats of dead animals; however, the feasibility has not been shown in lactating animals yet. Ring-like folds were present in the teat cistern, which protruded horizontally into the lumen. It was also possible to visualize the branchlike transition of the teat cistern into the larger milk ducts. Radiographic examination using barium sulfate contrast medium showed that the teat cistern ends in a network of initially wide but branching and narrowing milk ducts. The two teat canals and cisterns are completely independent of each other and there is no communication between the glandular tissue of the two canals and cisterns.

## Introduction

Dromedary camel was probably first domesticated in Somalia and Arabian Penisula, and generally is found in the semi-arid to arid regions, mainly in the Africa (Horn of Africa and Sahara Desert), Asia (Arabian Peninsula, but also Near East, and western and central Asia), and a significant feral population in Australia. In recent years camel milk production has gained increasing importance. Camels are well adapted to the dry and hot climate and under extreme climatic conditions, they can cope with little water and feed. In the 90s the idea of a camel milking facility was born at the Central Veterinary Research Laboratory (CVRL) in Dubai. With establishing of a dairy camel farm in Dubai, camel milk has been produced using modern agricultural methods.

The mammary gland of the dromedary consists of four quarters and four teats. Each teat has two, sometimes three teat canals (YAGIL 1985; WERNERY 2006). Each teat canal is associated to an individual glandular complex. The udder cistern is not a cavity like in bovine but rather a sponge like structure (WERNERY et al. 2008). Consequently, the camel udders typically consist of at least of eight separates, independent, milk producing units (WERNERY 2006, RIZK et al. 2017). The left and right halves of the udder are separated from each other by a double sheet of fibroelastic tissue extending from the *Linea alba* to prepubic tendon (DAMIAN et al. 2009). A groove is generally visible between the left and right halves of the udder. The cranial and caudal quarters are not differentiated macroscopically (DAMIAN et al. 2009, RIZK et al. 2017). The milk yield varies between the different dromedary breeds and between individual camels. As the milking and husbandry conditions on individual farms differ significantly there are major differences in the milk yield (FARAH and FISCHER 2004). In contrast to bovine the lactating dromedary camels need to have the stimulus of the calf during the entire lactation, otherwise milk production stops.

Mastitis in camels has been reported in camel-rearing countries worldwide (YOUNAN et al. 2001, MOHAMMED et al. 2005, HAWARI and HASSAWI 2008, WANJOHI et al. 2013, NIASARI-NASLAJI et al. 2016, JOHNSON et al. 2016). It is well known that mastitis is one of the most challenging diseases in high-yielding dairy cows (BANSAL and GUPTA 2009). Since dromedary camels have only recently been kept more intensively for milk production, there is less knowledge to which extent mastitis and udder injuries may lead to economic losses. However, as milk production increases the demand for modern diagnostic techniques increases concurrently. Ultrasonography, endoscopy and radiography has been used in bovines to evaluate udder health. Ultrasonography is a non-invasive diagnostic technique for the examination of mammary gland and teats which has also been applied to a limited amount in camels (ABSHENAS et al. 2007). Endoscopy is a well-established examination method for teat and the teat cistern in bovines (SHAKESPEARE 1998, VANGROENWEGHE et al. 2006). Additionally, teat endoscopy offers therapeutic possibilities. To our best knowledge there are currently no studies found on endoscopy and radiographic examination of the dromedary udder.

The objectives of this study were to evaluate the application of ultrasound and endoscopic technique to examine the teat cistern and the parenchyma of the dromedary mammary gland. It was also intended to visualize the structures of the individual gland complexes by radiographic examination. The first hypothesis of the study was that ultrasonography, radiography and endoscopy are suitable diagnostic tools for advanced camel udder examination. Secondary, it was hypothesized that by applying these techniques the specific anatomy of dromedary udders can be visualized and measurements can be taken contributing to establish physiologic reference ranges, which may be used for the selection of a milking dromedary.

## Material and methods

### Animals

The udders were removed from 49 female dromedary camels which were slaughtered in a local abattoir in Al Ain, United Arab Emirates (UAE). However, no data on the animals (e.g. age, lactation number, or calving date) were available. The udders were removed immediately after slaughter and transported to the Central Veterinary Research Laboratory (CVRL), Dubai, UAE in foam polysterol boxes on ice. After arriving in the laboratory, the udders were immediately macroscopically examined and marked with individual numbers. After that they were stored at 4 °C until further examinations. The udders were used for the x–ray, ultrasound and endoscopic examination. Due to changing availability of ultrasonographic, endoscopic and radiography devices not all udders could be examined applying all 3 methods.

Twenty-six udders were removed from female dromedary camels which were submitted for necropsy to the Central Veterinary Research Laboratory (CVRL) in Dubai, UAE. These animals were between three and 20 years old. All these dromedaries succumbed to different diseases, but none was reported to have any disease of the mammary gland. They were in different lactations stages or juvenile. The number of parities of the adult female dromedary varied widely. The udders were only used for macroscopic examination of the teats and teat canals.

Eleven freshly lactating dromedaries were available for ultrasonography of the udder. These animals were owned by the Camelicious (Emirates industry for camel milk products) farm in Dubai, UAE, between 14 and 21 years old and had an average of 4.2 lactations.

### Number of teat canals

The number of teat canals per teat was counted at all 86 examined udders (49 from slaughtered camels, 26 from camels admitted for necropsy, 11 lactating animals) by visual appraisal.

### Ultrasonography

#### Ultrasonography of udders from slaughtered animals

**Fig. 1:**
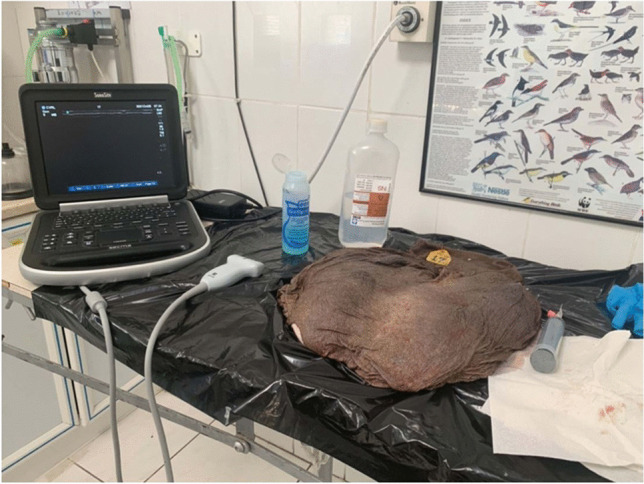
Workplace for the ultrasonographic examination of the dromedary udder from slaughtered animals

Thirty two of the 49 udders from slaughtered animals were examined using an ultrasound device (Sonosite, Secma medical innovation, Skaevinge, Denmark). The udders were placed teats facing up on a flat surface (Fig. 1). The teats and the udder were examined with the B-mode real time ultrasonography with the Sonosite HFL 50 × 15–6 MHz multi-frequency, broadband, 50 mm linear array transduce.

**Fig. 2:**
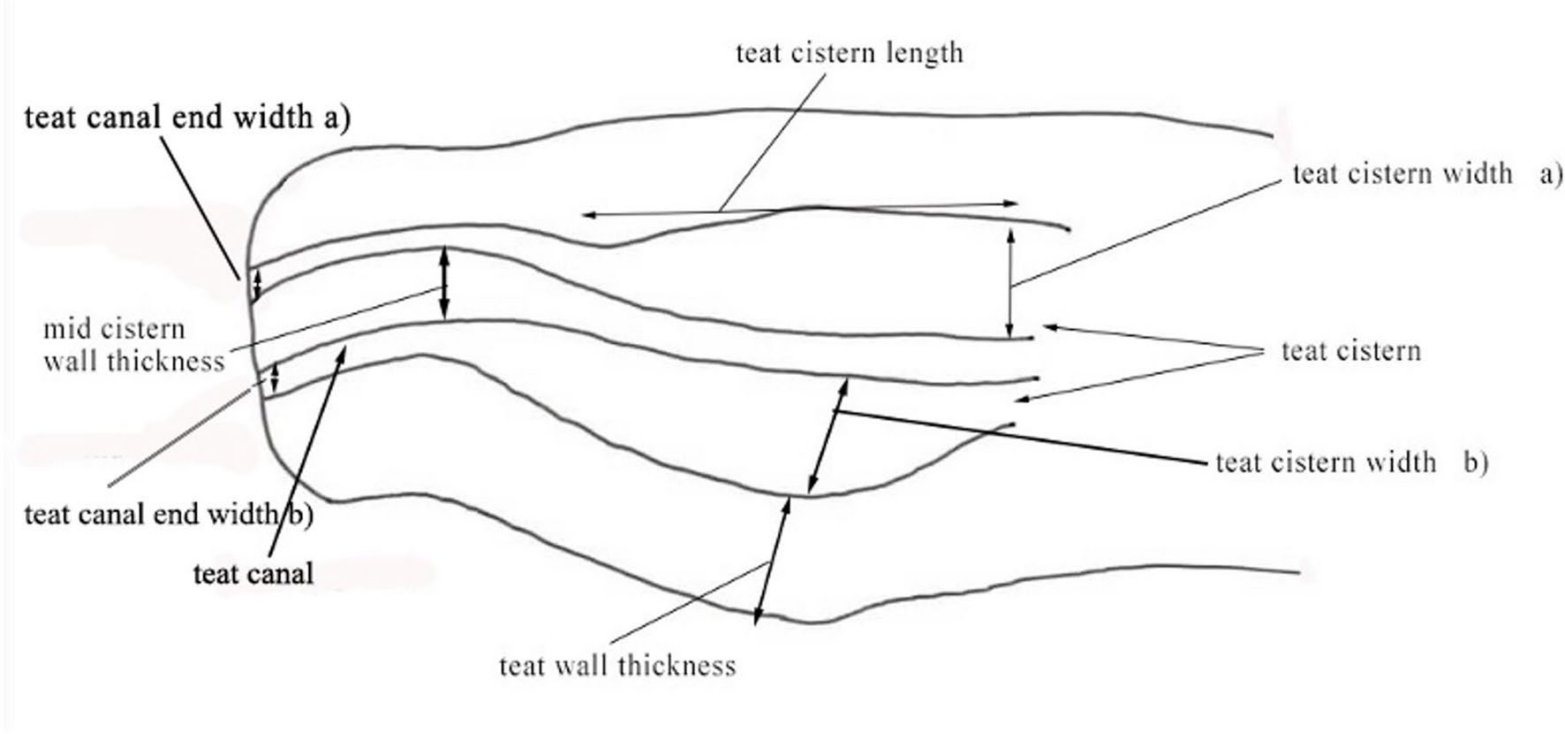
Measurements of the camel teat taken using ultrasonography

Sterile saline solution was injected into each teat canal through indwelling catheter. The teats were filled with the saline solution until completely filled. After that, ultrasound gel was applied to the udder and teats. The examination was carried out sagittally from the base to the tip of the teat, followed by a longitudinal examination of the teat. The following ultrasonographic measurements (Fig. 2) were taken from 128 teats:

- Teat cistern length
- Teat end width (front and hind teats, left side and right side)
- Teat wall thickness
- Teat cistern width (front and hind teats, left side and right side)
- Mid cisternal wall thickness

Images of the measurements were stored on a USB drive.

#### Ultrasonography in lactating animals

Eleven lactating dromedaries were available for ultrasonographic examinations.

**Fig. 3:**
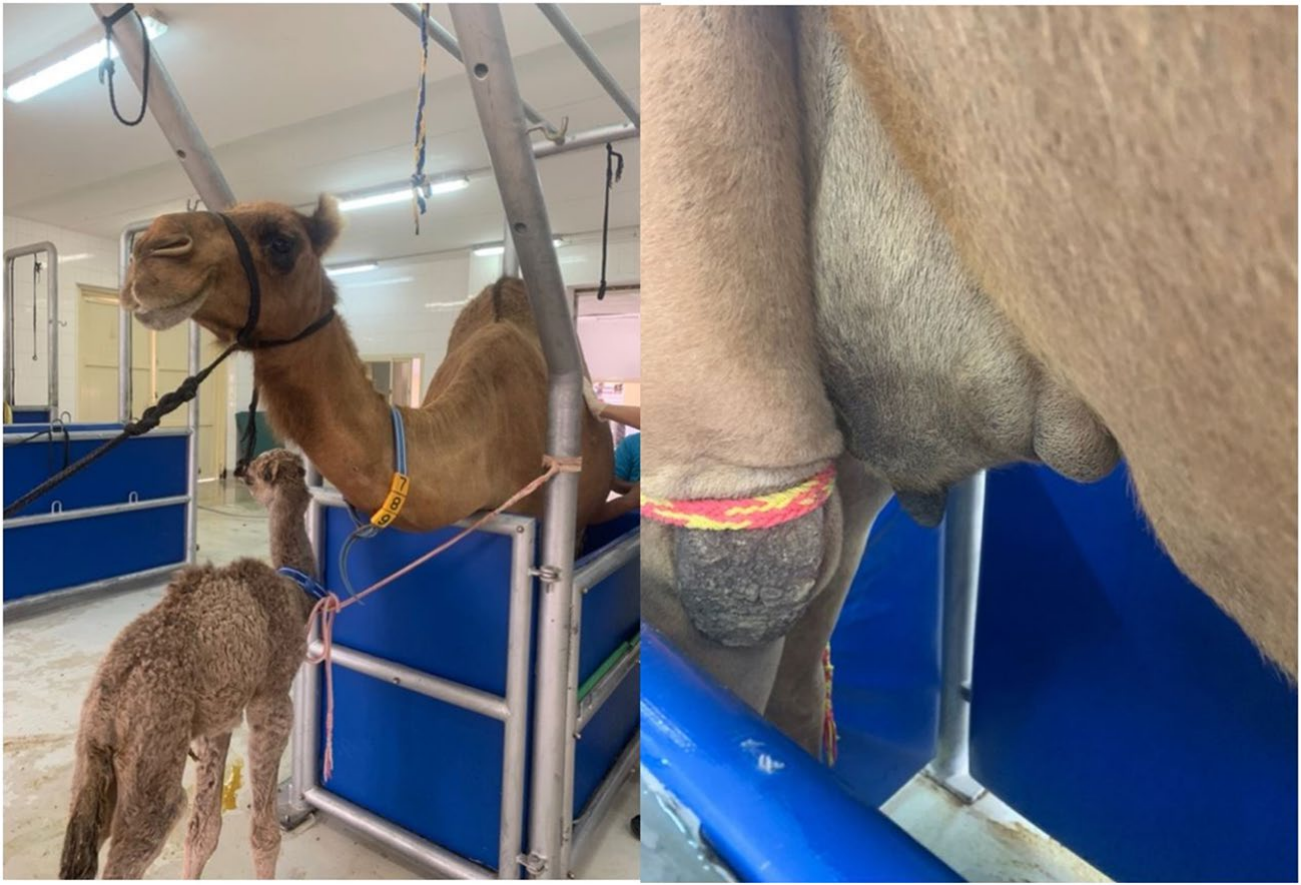
Examination chute for the ultrasonographic examination, showing also the fixation technique of the animal during ultrasonography

The animals were restrained together with their calves in an examination chute (Fig. 3). The head was fixed with a halter and a rope. To ensure the safety of the examiner, the hind legs were tied together with a rope to prevent kicking (Fig. 3). The ultrasound of the teats was performed with a water-filled cup as described for cattle (FRANZ et al. 2009). Sagittal images were taken from the tip, middle and base of the teat. Furthermore, longitudinal pictures were taken of each teat. The ultrasound device (ALOKA SSD–500, Mulyani Medical, Jakarta, Indonesia) used was connected to a printer (Sony video graphic printer UP–895MD). Due to the stimulation of the udder, in most animals the milk started dripping very quickly, therefore it was decided to limit the measurements to the parameters listed below. The images were then scanned and stored digitally.

The measurements of the following parameters (Fig. 2) were measured:

- Teat wall thickness (front and hind teats, left side and right side
- Teat cistern width (front and hind teats, left side and right side)
- Mid cisternal wall thickness

### Endoscopy

The teat cistern and the transition into the teat canal were examined endoscopically in 12 randomly selected udders of the 49 slaughterhouse udders. For the examination a rigid endoscope (Karl Storz serial number 22201020, 20,133,120 and WD 250, Tuttlingen, Germany) with the lens system Image 1 HD (2,222,050) was used. The endoscope had a diameter of 2.7 mm, a length of 18 cm and an angle of 30°. The images were saved to a USB drive. The teats were inflated with air by the built in compressor of the endoscope system. The endoscope was inserted into each teat canal. The video recording was started immediately after insertion into the teat canal and ended after complete examination, when removing from the teat canal.

### Radiography

**Fig. 4:**
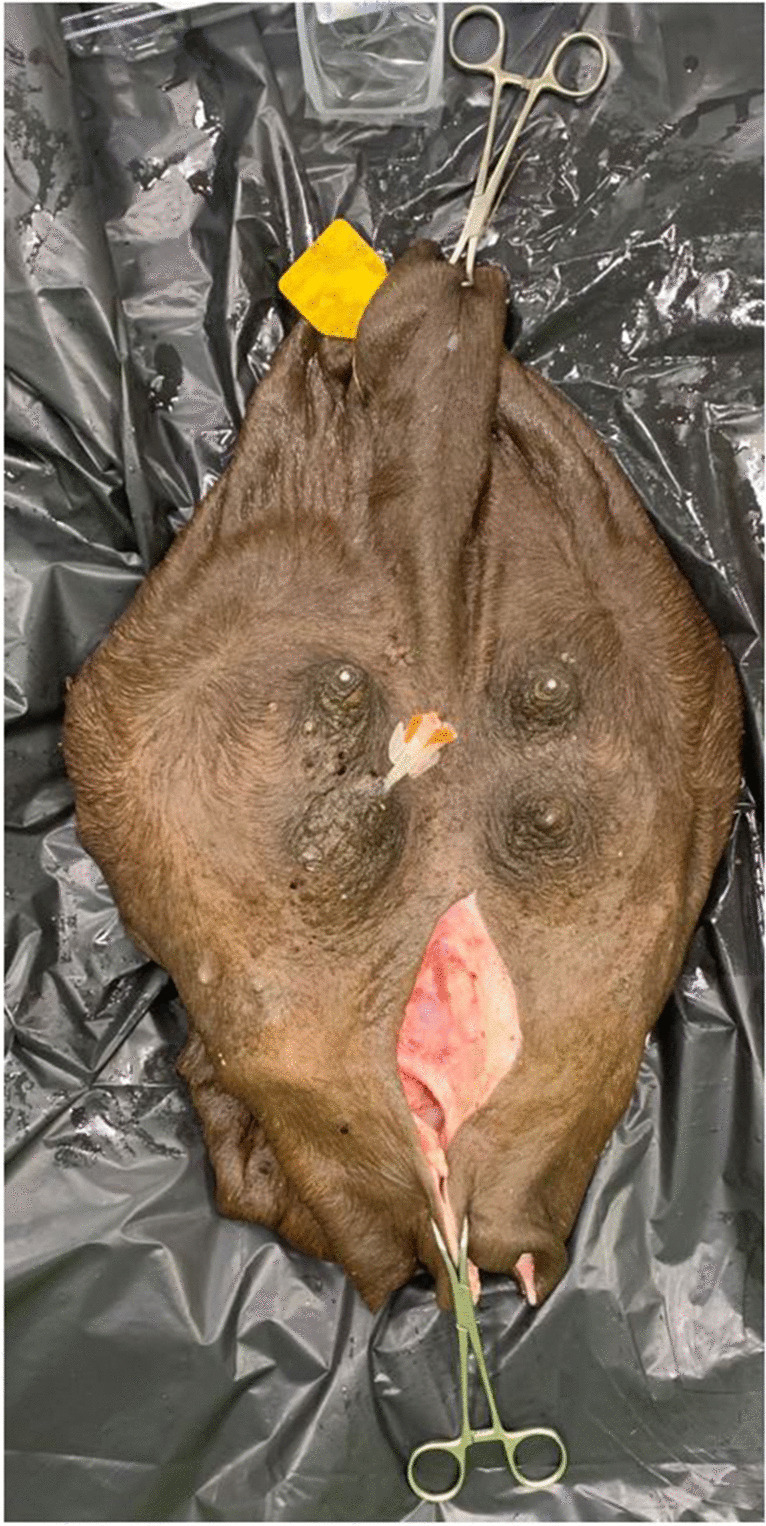
Preparation of the udder before radiography, view from ventral

Eleven of the 49 udders from slaughtered animal were examined radiographically. Each udder was placed flat on a flat surface with teats up. To visualize the internal structures of the udder halves, indwelling vein catheters (TERUMO SURFLO I.V. Catheter 16 G × 2″, Tokyo Japan) were inserted into each ostium papillae (Fig. 4). Barium sulfate (Vet-Way Barium sulphate, Elvington, York, UK) solution (1:2) was instilled in only one teat canal per teat to avoid overlapping. The injected volume was based on the respective size of the udder halves. The indwelling vein catheters were then closed with surgical clamps and the udder was hung simulating a physiological position with additional surgical clamps and positioned in front of the radiographic plate (Canon CXDI-801C Wireless, Irvine, USA).

A latero–lateral image was taken with closed catheters, afterwards another image with catheters open to get a higher contrast image. Subsequently, the barium sulfate solution was injected into the second teat canal in the same teats and an identical procedure to the first was carried out. The radiographs were taken with the Xprime Vet-20BT machine (Poskom, Gyeonggi-do, South Korea). Only one half of the udder was radiographed to avoid overlapping. The images were digitally stored on the radiograph machine.

### Statistical analysis

The statistical evaluation was carried out using IBM SPSS Version 29. Data was checked for normal distribution using the Kolmogorov–Smirnov test and then quartiles and standard deviation were calculated. Since normal distribution was not given, log transformation was applied and mixed model analysis was used for the comparisons between right and left, front and hind, and lactating and dead. *P* < 0.05 was considered significant.

## Results

### Numbers of teat canals

Table 1 gives an overview of the distribution of the number of teat canals in 86 examined udders. One female dromedary from 26 animals delivered for necropsy, had three canals on the left front teat. One had three canals on the right front teat, one had three canals on the right hind teat and one dromedary had three canals on both hind teats. From the 49 slaughterhouse udders there were three dromedaries with three canals in the left hind teat. Five dromedaries had three canals in the right hind teat. One animal had only one canal per teat on the left front and left hind teat. From the 11 lactating female dromedaries used for ultrasonography, one animal had three canals in the left hind teat.

**Table 1:** Overview of the distribution of the number of the teat canals in 86 examined udders

	Left: Number/Percentage	Right: Number/Percentage
Front	One canal: 1/1.16% Two canals: 84/97.67% Three canals: 1/1.16%	One canal: 0 Two canals: 85/98.84% Three canals: 1/1.16%
Hind	One canal: 1/1.16% Two canals: 81/ 94.18% Three canals: 4/4.65%	One canal: 0 Two canals: 79/ 91.86% Three canals: 7/8,14%

### Ultrasonographic examination

Ultrasonography of teats and glandular tissue was performed on 32 slaughterhouse udders (128 teats) and 11 lactating animals (44 teats) on the Camelicious Farm (UAE) were examined.

**Fig. 5:**
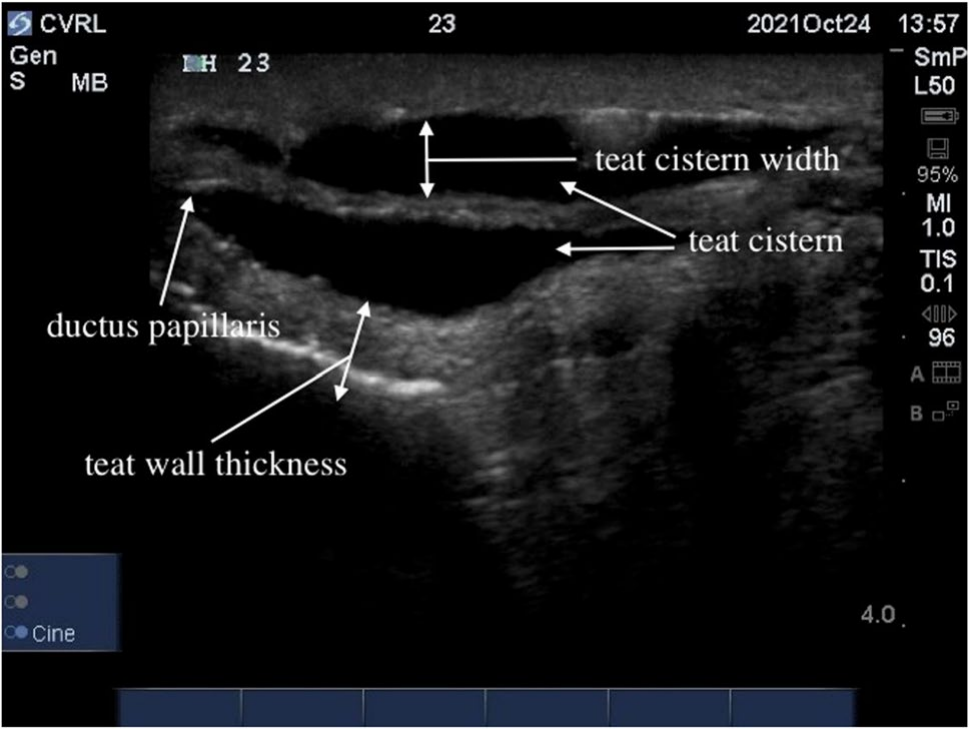
Sagittal scan of the two teat cistern in an udder of a slaughtered camel

**Fig. 6:**
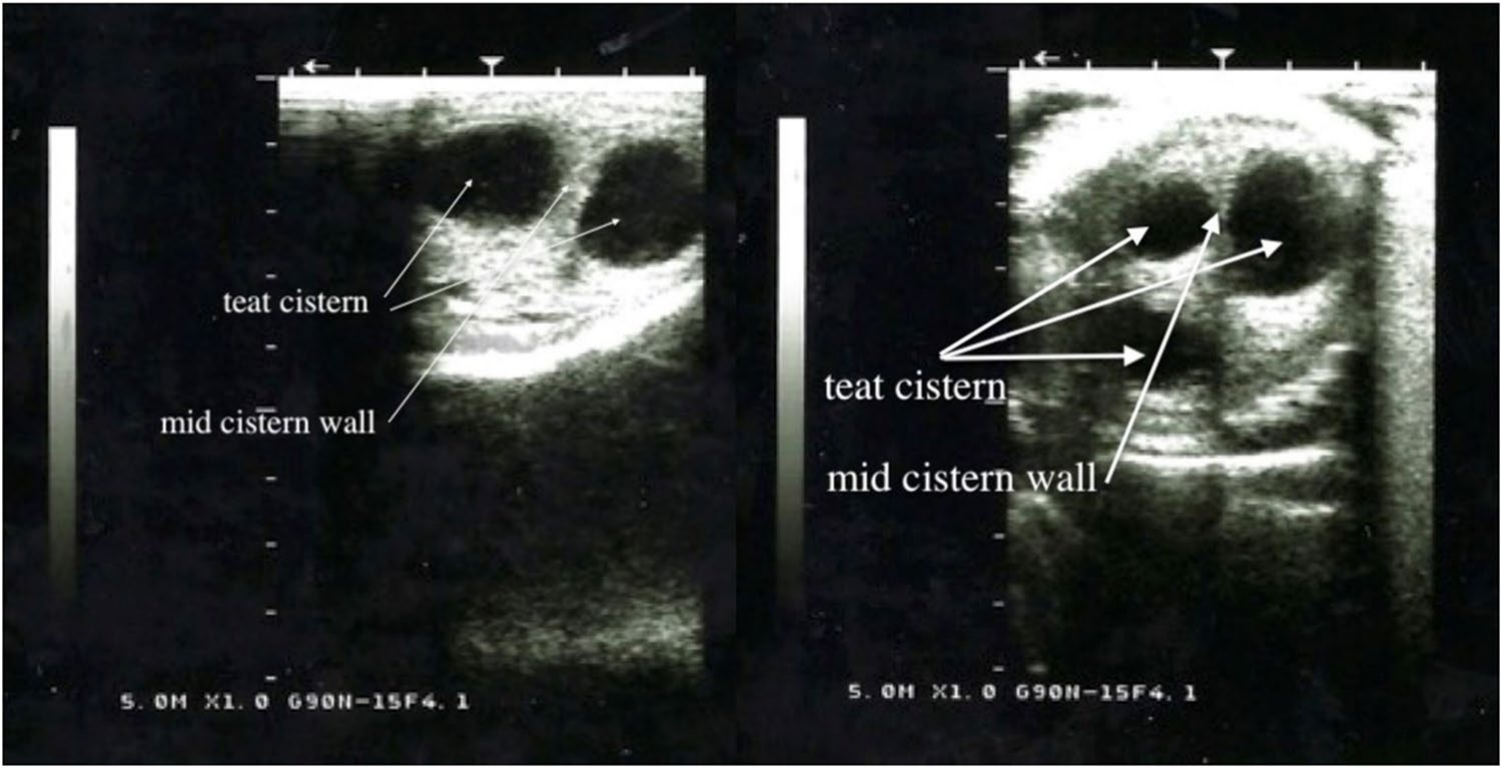
Left: Transverse scan of the two teat cisterns of a lactating camel, Right: transverse scan of three teat cistern of a lactating camel

**Fig. 7:**
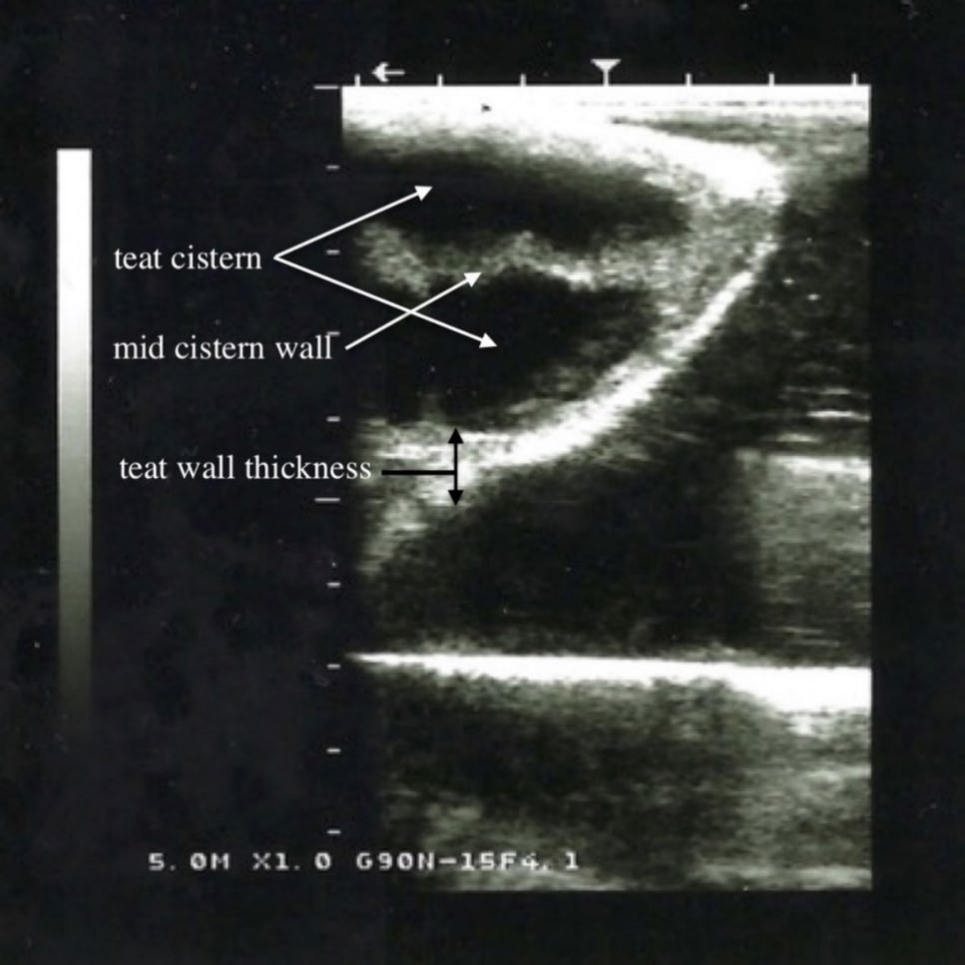
Sagittal scan of the two teat cisterns of a lactating camel

**Fig. 8:**
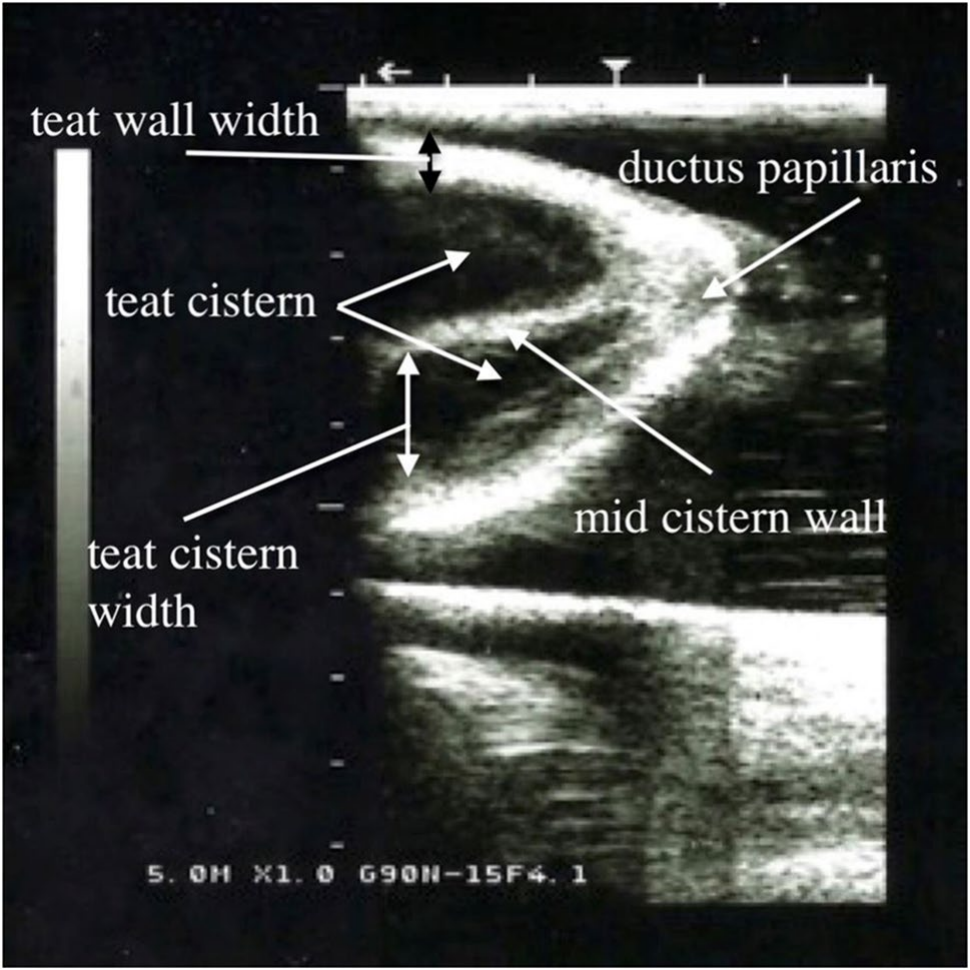
Sagittal scan of the two teat cisterns of a lactating camel

In the sagittal scan the teat canal consisted of three layers, a hyperechoic outer layer (skin), a thick hypoechoic middle layer and a more echogenic inner layer. The water filled lumen of the teat cistern was anechoic (Figs. 5, 6, and 7). In the lactating animals the lumen is also predominantly anechogenic but contained small echogenic particles in the lumen of the teat cistern in some animals. Between the two teat canals was a hyperechoic layer was visible (Figs. 5, 6 and 7). Both teat cisterns appear to have the same size in the lactating animals (Figs. 6 and 8). The teat canals appeared as a thin anechoic line bordered on each side by an echoic thicker layer.

A hypoechoic homogenous granular structure could be visualized in the udder parenchyma of the udders from the slaughtered animals. The lactiferous ducts were anechoic. In udders with larger lactiferous ducts, the milk was anechoic or contained some small echogenic particles. The measurements of the dromedary udder of the slaughtered and lactating camels are summarized in Tables 2 and 3.

**Table 2:** Ultrasonographic measurements of the mammary glands of slaughtered camels

	Front quarters			Hind quarters		
	1. Quartil (mm)	Median (mm)	3. Quartil (mm)	1. Quartil (mm)	Median (mm)	3. Quartil (mm)
Teat cisternal length	3.29	4.16	4.64	2.68	3.78	4.49
Teat end width left	0.14	0.17	0.22	0.15	0.19	0.24
Teat end width right	0.12	0.15	0.22	0.13	0.15	0.18
Teat wall thickness	0.31	0.39	0.55	0.32	0.4	0.56
Teat cistern width left	0.49	0.81	1.27	0.48	0.78	1.19
Teat cistern width right	0.41	0.69	0.97	0.4	0.67	1.06
Mid cisternal wall thickness	0.19	0.29	0.45	0.2	0.25	0.38

**Table 3:** Ultrasonographic measurements of the mammary glands in lactating camels

	Front quarters			Hind quarters		
	1. Quartil (mm)	Median (mm)	3. Quartil (mm)	1. Quartil (mm)	Median (mm)	3. Quartil (mm)
Teat wall thickness	0.3	0.45	0.6	0.4	0.5	0.6
Teat cistern width left	0.42	0.65	1.27	0.8	1.0	1.4
Teat cistern width right	0.52	0.7	1.0	0.6	1.0	1.4
Mid cisternal wall thickness	0.2	0.2	0.37	0.2	0.2	0.3

The measured parameters were not normally distributed. When comparing front to hind quarters and left to right udder halves, no significant differences were present. Comparing udder measurements from slaughterhouse material to lactating animals, no significant differences could be found.

### Endoscopic examination

**Fig. 9:**
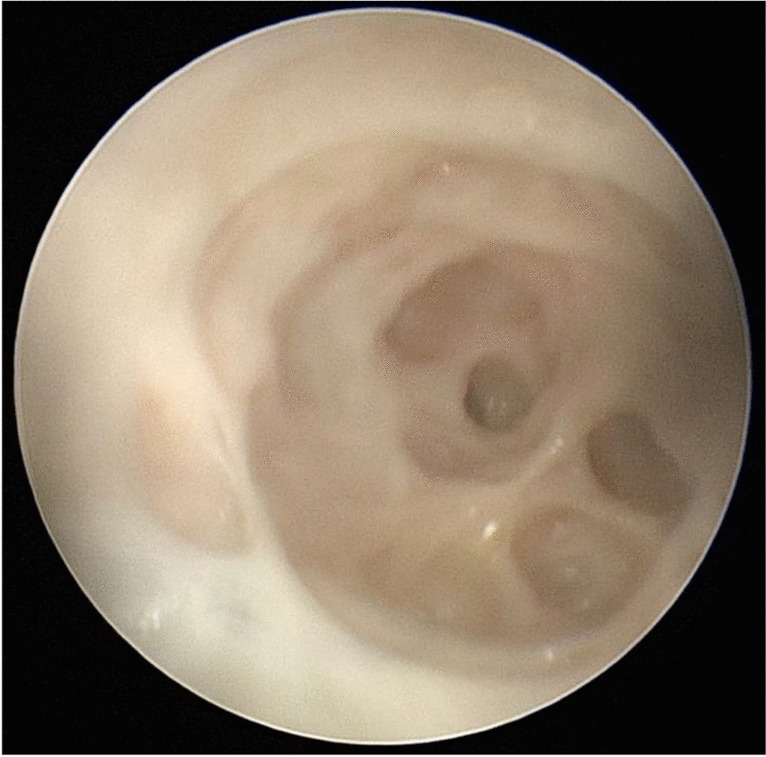
Endoscopic examination of the dromedary camel udder. View from the teat cistern on the spongy structure of the glandular part continuing into the milk ducts

Endoscopy was performed in mammary glands from 12 slaughtered animals. Ring-like folds were present in the teat cistern, which protruded horizontally into the lumen. At the transition from the teat cistern into the glandular part numerous canals that branched off into smaller canals were visible (Fig. 9). Due to the use of a rigid endoscope, it was not possible to follow the branches further proximally. There was no obvious difference between the front or hind teats during the endoscopic examination.

### Radiographic examination

Radiographic examinations were performed in mammary glands of 11 slaughtered camels.

On average 49 ml of barium sulphate solution was injected into each teat canal. The minimum amount was 30 ml and the maximum amount was 118 ml. To obtain better images, the catheter was opened shortly before the radiograph was taken.

**Fig. 10:**
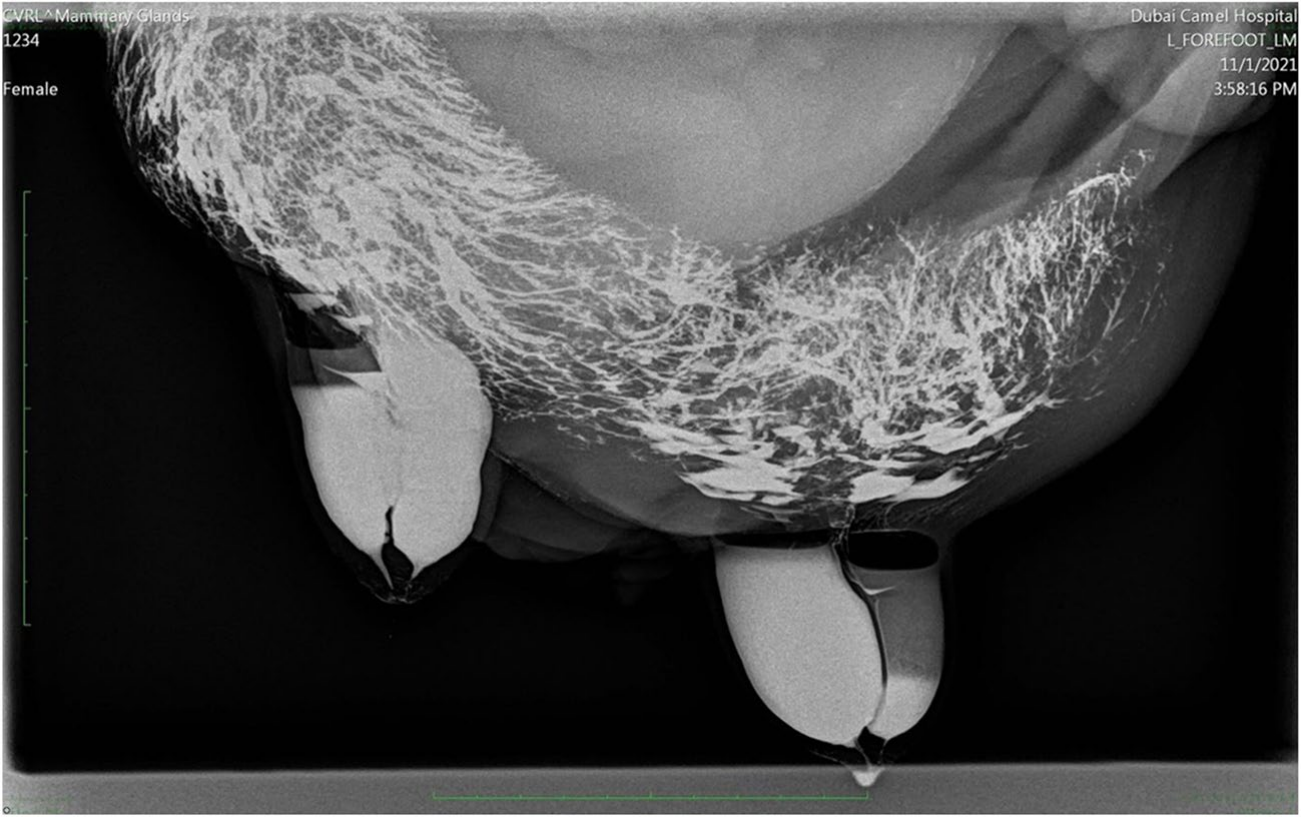
Radiographic examination of the dromedary udder in a latero-lateral image

All examined udders showed two teat canals and milk producing complexes per teat. The teat cistern is followed by a network of wider into narrow ducts (Fig. 10). The two teat canals were independent of each other and there was no communication between the glandular tissue of the two canals.

## Discussion

The aim of the present study was to explore the application and feasibility of ultrasonography, endoscopy and radiography as diagnostic tools for the udder examination in dromedary camels. All three techniques can apply in these animal species for visualized the specific anatomy of dromedary udder and measurements can be taken contributing to physiologic reference ranges, which may be used for the selection of a milking dromedary and detection and treatment of udder diseases. Eighty-six dromedary udders were measured and examined.

One objective of this study was to describe ultrasonographic appearance of healthy mammary glands in dromedary camels as prerequisite to detect pathological changes. Since dromedaries are kept more frequently as dairy animals, udder health plays an increasingly important role for an economical and hygienic milk production. Hence, advanced diagnostic techniques are likely to gain importance.

It has been shown that ultrasonography of the udder is a non-invasive technique, which can be easily performed in lactating animals if restrained properly. The frequency and transducer type in the present study were feasible to examine all portions of the gland parenchyma and teats. As described for cattle by FRANZ et al. 2001, the teat of the lactating animals was submersed in a waterfilled cup, but it was also possible to perform the ultrasonographic scanning directly. Nevertheless, the quality of images was superior when the teat was examined in a water bath. Additionally, the teat wall as well as the Furstenberg area cannot be visualized completely without using the filled water cup. Since the teats of the slaughtered camels were filled with water it was not necessary to do the ultrasonography in a water bath.

The ultrasonographic findings showed a complete separation between the two or three teat cisterns. The ultrasound images correspond to those of other animal species like for example cows, horses and goats, showing a three-layer with two hypoechoic teat cisterns in the middle. ABSHENAS et al. (2007) described defined gland and teat cisterns are present in camel udders. VYAS et al. (2019) also showed, using ultrasonography, the presence of definite teat and gland cisterns. In contrast to cattle, dromedary udders do not have mammary gland cisterns. The teat cistern continues in a branch of larger milk ducts subsequently branching out more and more finely. These findings corresponded with our results of contrast radiographic and endoscopic examination.

The ultrasonographic measurements of the teat cisternal length, teat end width, teat wall thickness, teat cistern width and mid cisternal wall thickness showed a wide variability in individual camels. Even in comparison with other authors there were also wide variabilities in these parameters (SALEH et al. 1971 and ABSHENAS et al. 2007). SALEH et al. (1971) showed that both front and hind teats are almost equal in length, this is similar to our findings. But they also showed that there is a huge range between the individual camel udders. ABSHEENAS et al. (2007) found no statistically significant difference (*p* > 0.05) between front and hind parameters of the measured camel udders. This may be due to the lack of selection during breeding of the dromedary for milk yield and lactation status. The milk ejection may have also influenced the measurements. EBTSAM et al. (2020) described the parenchyma of a healthy mammary gland of a goat as a homogenous structure of average echogenicity filled with anechoic content (milk). FRANZ et al. (2009) also describe the udder parenchyma of cows as a uniformly echogenic structure. The same was seen in horses during ultrasonographic examination (ENNEN et al. 2011), correlating to our findings in dromedaries. In the sagittal scan the teat canal consisted of three layers, a hyperechoic outer layer, a thick hypoechoic middle layer and a more echogenic inner layer. The lumen of the teat cistern was anechoic. This also corresponds to the results for cattle with the exception that there is only one teat canal or teat cistern (FRANZ 2011).

The second aim of the study was to explore to which extent endoscopy and radiography are feasible additions of examinations of the camel udder.

The endoscopic examination revealed a very simple and practicable method for examining the inner structures of the teats, using a rigid endoscope. It was possible to explore the teat and teat cistern and the branching of the milk ducts. However, it was not possible to penetrate further into the individual branches of the glandular tissue. In their study VANGROENWEGHE et al. (2006) described problems in visualizing the internal structures of the bovine udder caused by the flow of milk. We only used dromedary udders from slaughtered animals without any milk content in our study and inflated them with air for better visualization, but it can be assumed that the problem would occur during endoscopy of the udder in lactating camels.

GEISHAUSER and QUERENGAESSER (2001) described benefits of using endoscopy to diagnose and treat milk flow disorders in cows. They found that it is much more efficient to use an endoscope than to proceed blindly. It might be an option to take a flexible endoscope. This may cause less damage to the teat mucosa and it would be possible to penetrate further into the milk ducts. It could be a problem with a flexible endoscope which may bend. Further examinations on lactating camels must be carried out to find out whether this is also a problem in dromedaries. However, in conclusion from the present study it seems very likely that similar to cows, it will be possible to use the minimal invasive endoscopy techniques for diagnosis and therapy in camel udders.

Radiography is a non-invasive examination, just like the ultrasonographic examination, but it is much more difficult in terms of technical complexity. Using radiography, the teat cistern drain and the transition in the ducts of the gland was visualized by administration of contrast medium. To our best knowledge it is currently unknown if it is possible or practicable to inject contrast medium into the teat of lactating animals. To what extent a native image is sufficient for assessment must be clarified by further studies. However, images without contrast medium do not seem to be interpretable due to overlapping of soft tissue individual udder layers.

## Conclusions

The results of our examination showed that the ultrasonographic examination is a feasible tool for non-invasive diagnostics as well suitable for diagnostics in non-sedated animals. The examinations of camels retrained in a chute were easy to perform. However, additional examinations are necessary to evaluate pathological ultrasonographic images of the udder and teat of camels.

Endoscopic examination seems to be a potential diagnostic tool for internal examination of the teat canal and teat cistern. However, the feasibility has to be more thoroughly investigate in lactating animals. Radiography, in contrast, cannot be considered practical and will unlikely lead to more detailed results using contrast medium, for the visualization of internal udder structures. However, it does not allow interpretation due to the overlapping soft tissue. Radiography seems to be least feasible as diagnostic tool for camel udder and teats.

## Data Availability

The datasets generated during the current study are not publicly available.
